# Clinical Characterization and Prediction of Bipolar Disorder Evolution

**DOI:** 10.3390/jcm14072159

**Published:** 2025-03-21

**Authors:** Petr Kloucek, Armin von Gunten, Sylfa Fassassi

**Affiliations:** 1 SUPAA, Hôpital de Cery, Route de Cery, CH-1008 Prilly, Lausanne, Switzerland; armin.von-gunten@chuv.ch; 2 Service de Psychiatrie Générale, Site de Cery, Lausanne University Hospital, Route de Cery, CH-1008 Prilly, Lausanne, Switzerland; sylfa.fassassi@chuv.ch

**Keywords:** bipolar disorder, actigraphy, fractal dimension, Hurst exponent, stochastic optimization

## Abstract

**Background**: This paper addresses the possibility of replacing subjective evaluations of mental disorders with analytical tools based on large data provided by wearable sensors in combination with subsequent complexity mesoscale data projection using constitutive mathematical frameworks. **Methods**: The presented methods are based on the combination of a complexity/fractal approach and stochastic optimization, yielding Digital Mental Biomarkers (DMBs). **Results**: Analytic indexing can effectively augment the Young Mania Rating Scale, DSM-5 criteria, or structured interview diagnostics. The analytical approach allows us to carry out a prediction of mental disorder evolution as well as a subsequent probability characterization of BD episode progression over time. **Conclusions**: The presented analytical framework presents a semicontinuous diagnostic tool in the area of mental disorders, specifically applicable to bipolar disorder with corresponding manic episode indexing.

## 1. Introduction

We present conceptual analytical biomarkers capable of differentiating and predicting acute phases (manic, mixed, or depressive episodes) from transitional phases and euthymic periods in people with bipolar disorder (BD). Ultimately, we expect this to allow us to predict both the clinical course and early diagnosis in recurrent depression secondary to BD as opposed to recurrent depression unrelated to BD. Predicting the clinical course of BD would also allow us to conduct anticipatory reliable monitoring of therapeutic responses.

### 1.1. Bipolar Disorder

BD is a chronic mood disorder characterized by a combination of manic and depressive episodes over time. BD is a disabling condition [1] that usually starts in early adulthood. A late diagnosis worsens the prognosis, causing an increase in complications, the appearance of comorbidities, and/or resistance to the prescribed treatment. A large body of evidence shows that there is still an excessive delay in the accurate diagnosis of BD. Clinical research should ideally make it possible to better define BD in order to distinguish, during a depressive episode (i.e., often the first manifestation of the disorder), whether it is a bipolar or unipolar disorder. This could help reduce the taxonomic delay conceptualized by K. Fritz et al., [2], such as the lack of the detection of the disease once the first symptoms have occurred. Scientific studies should also help reduce the gaps in our knowledge or our inability to define the early clinical presentation(s) of BD before the emergence of an episode, which could prevent delays in detection [2].

Hypomania or mania, once they occur, are the only markers currently available that can be used to diagnose BD. However, BD is a complex disorder of affective, cognitive, and behavioral symptoms and signs evolving over time. Diagnosis today is based on combinations of subjective and objective features that accompany various phases of BD, with little or nothing at hand to accurately predict incipient changes in the various phases of BD. The predictive unreliability of subjective symptoms suggests searching for objective parameters, allowing for the prediction of temporal changes in clinical BD manifestations. Incipient sleep and activity pattern changes may be among the more useful markers that can be used to predict phase changes in BD. Indeed, activity is one of the key elements of bipolar symptomatology that needs to be interpreted from a dynamic perspective, allowing us to recognize the complexity of human physiology and rhythms. Recent changes in diagnostic criteria for BD have recognized the key role of activity in the symptomatology of BD. This is worth considering because, unlike mood, which we measure with clinical scales, activity can be measured objectively. It is also important to note that mood variations associated with BD may undergo semi-chaotic dynamics [3].

Clinicians use standardized clinical scales, e.g., DSM-5 criteria (American Psychiatric Association. Diagnostic and Statistical Manual of Mental Disorders (DSM-5) (2013)), and structured interviews [4] as diagnostics. Replacing clinical (statistical) examinations with analytical complexity tools has the potential to guide clinicians toward performing earlier interventions, resulting in treatments that are more effective and often result in fewer side effects.

**Figure 1:**
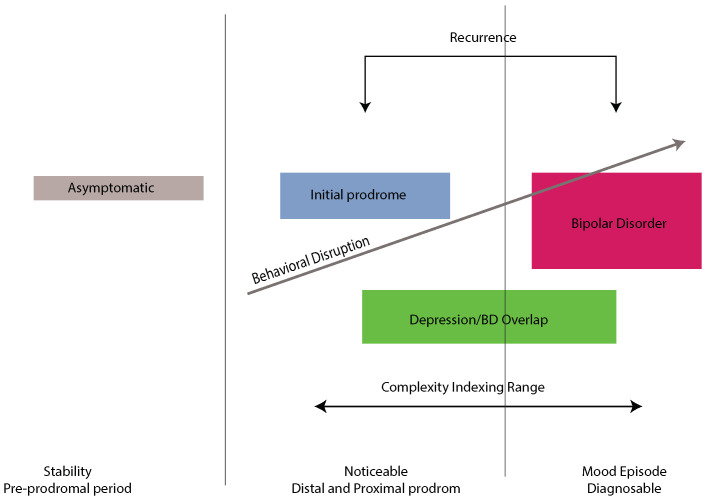
The three distinctive regions of evolution. The application of the complexity/fractal mathematical approach allows us to not only quantitatively index BD but also to introduce BD evolution prediction, as well as global and resistance indices (not discussed in this paper). A mathematical approach can distinguish between depressive episodes covering BD that can be seen beneath the depressive layer.

The difficulties of treating major depression and bipolar depression, as well as recent advances both in clinical guidelines and in pharmacological treatment, can be found in [5,6,7]. One possible problem is overshadowing of BD by an observable depression phase, c.f., Figure 1.

### 1.2. Application of Actigraphy

Actigraphic data are usually transferred to a computer and analyzed using software. These mathematical theories can translate movement into activity counts over predefined durations, called "movements per epoch". Actigraphy uses customizable activity thresholds to distinguish between different intensities of activities and sleep. Scoring is often automated by mathematical models. Different variables are extracted from self-similar actimetric data: (i) sleep variables (timing, duration, etc.); (ii) activity variables (amount of activity, mean activity, etc.); and (iii) rhythm variables (period, amplitude, and phase) [8].

Actigraphy is a non-invasive method of monitoring human rest–activity cycles, particularly during BD manic episodes. The analysis of the actigraphic data of a manic patient can provide several critical pieces of information about a patient’s sleep–wake patterns. Traditional linear analyses of actigraphy data in manic patients face several limitations, including sensitivity to erratic activity patterns and an inability to capture multidimensional changes that can be observed in manic episodes. The complexity projection method presented provides a more comprehensive picture of what a patient’s condition might encompass.

Activity analytics with the subsequent mesoscale projection of microscopic sensor data lends itself as a suitable digital biomarker to follow over time. The objective measurement of activity using an actigraphic device is now an accepted method [9].

## 2. Methods

The presented method focuses on the usage of wearable sensors and a subsequent coarse-graining of large amounts of microscopic equidistant self-similar time sequences with (multi-)fractal structures. The complexity/fractal mesoscale/medical projection using the Hurst exponent [10,11,12,13] allows for analytic indexing replacing, e.g., Young Mania Rating Scale indices. It allows the prediction of mental disorders using a stochastic approach as well as probability characterization of BD episodes.

The Hurst exponent can be interpreted as a probability measure of a decay of time-series autocorrelation. The decay indicates that an observed signal is losing its repetitive pattern, i.e., a delayed copy of itself. Thus, decay of autocorrelation can be considered to be an increasing absence of “memory”, characterized by an absence of predictable behavioral patterns. Moreover, such decay also exhibits itself as a lack of a measured response to both external and/or internal stimuli. We hypothesize that the presence of a mental disorder can be detected and quantified by the time evolution of complexity surrogate signs of partial behavioral patterns autocorrelation in high-dimensional spaces modeling physiological, behavioral, topological, and environmental influences. Accepting this hypothesis, we conjecture that various mental disorders differ by their complexity/fractal dimension.

The presented approach, applicable, in general, to various objective diagnoses of mental disorders, combines longer time acquisition of surrogate data with subsequent mathematical coarse-graining of the raw input using complexity projections as well as stochastic optimization to achieve short-term predictability.

The first focal point is the introduction of *Digital Mental Biomarkers* (*DMB*). Specifically, the complexity projections of bio-sensor-generated time sequences open possibilities for quantitative re-definition of the Young Mania Rating Scale (YMRS), c.f., Section 2.2.

The second focal point concerns the predictability of mental states, particularly BD manic episodes. This possibility has not been explored so-far to our best knowledge. Our approach is based on finding the best fit of mono-fractal Gaussian white-noise approximation of a given complexity projection of, possibly, multifractal microscale vital sensory data, c.f., Section 2.3.

Thirdly, we are proposing a way to evaluate the probability of stable, meta-stable, and observable BD episodes.

The mathematical approaches to the above two areas are described in some detail in Section 3.

### 2.1. Limitations

Several issues are not addressed in the presented communications.

1. The paper does not provide a broad longitudinal study of the applicability of accelerometer-based measurements related to BD, as this is well established in the literature. Nevertheless, the patient data used in this communication are based on 20 weeks of continuous measurements representing about 20,000 time data points.
2. The paper is solely focused on the application of complexity projections of sensory data and the possibility of providing objective evaluations of BD episodes, their probability of appearing, as well as their predictability. Consequently, this study focuses on novel techniques rather than comparative analysis with existing methods. We chose a patient, code name C207, as an example to demonstrate the presented phenomenology and methodology.
3. The efficiency of the presented approach is mentioned only briefly in Section 2.2 as it is not the main focus of the presented paper.

### 2.2. Digital Mental Biomarkers

**Figure 2:**
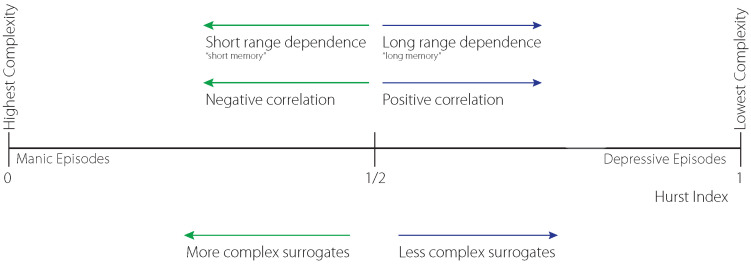
A possible interpretation of the Hurst exponent, “fractal dimension” in the Mental Disorders framework. Short vs. long (pattern) dependence characterizes low vs. high probability of behavioral repetitions.

The origin of the presented Digital Mental Biomarkers is rooted in the autocorrelation of repetition of various (past) time-sequence patterns. This autocorrelation is expressed by the Hurst exponent, i.e., the Hasdorff–Besicovitch dimension of fractal structures of self-similar sequences. The underlying complexity (low probability of repetition) and its interpretation as “memory” is shown in Figure 2.

The fractal scale is represented by the Hurst exponent (*H*), with a range (0, 1). The exponent is important because it characterizes persistent, positively correlated, i.e., “longer memory” , sequences if H>1/2 and negatively correlated, anti-persistent sequences otherwise. A Wiener process is characterized by H=1/2 yielding neither persistent nor anti-persistent behavior represented by surrogate data.

It is important to mention that *H* is related to fractal dimensions by the formula d−H, *d* being the dimension of the ambient space in the case of self-similar time series.

The complexity representation of vital time sequences removes the physical units that are present in vital surrogate sensor data. This feature is fundamental when analyzing different time sequences.

**Figure 3:**
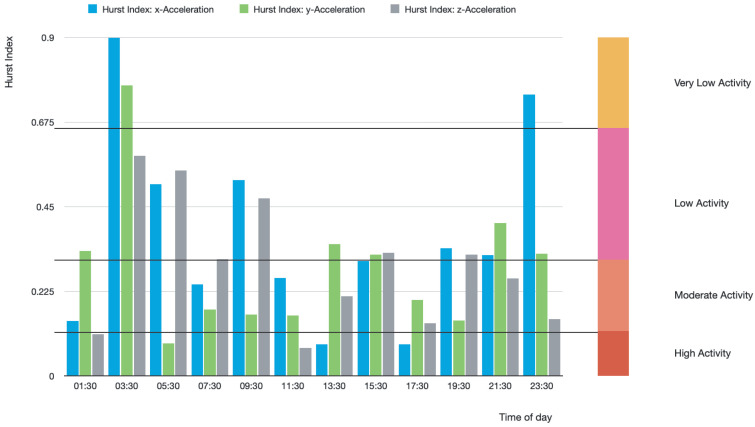
A graphical visualization of the approach outlined in Section 2 using a complexity projection of 3D*X*, *Y* and *Z* axes right wrist accelerometer data of the patient C207 with clinical BD diagnosis represented by different colors.

An application of the presented complexity projection approach is shown in Figure 3 and Table 1 using the acceleration of the left hand of a BD patient in three dimensions. The *H* values below $\frac{1}{2}$ indicate rather erratic hand movements corresponding to an anti-persistent state that can be possibly interpreted as “manic state ” which is negatively correlated.

While the Hurst exponent provides valuable insights into the predictability of rhythm, it is essential to consider its limitations. The calculation of the Hurst exponent assumes stationarity and fractal properties, which may not hold true for all types of time-series data. Additionally, the interpretation of the Hurst exponent should be context-dependent and complemented by other analysis techniques. Understanding the specific characteristics and context of the rhythm being analyzed is crucial to applying the Hurst exponent effectively, c.f., Table 2.

A clinical application of the proposed methodology is mentioned at Appendix A.

#### Global View of the Complexity Projection of Acceleration Data

The Hurst exponent is particularly useful in understanding the underlying structure and predictability of complex rhythmic patterns. Rhythmic patterns often display fractal properties, meaning that they exhibit similar structures and behaviors across multiple scales of observation. By calculating the Hurst exponent, we assess the probability of a rhythm exhibiting persistent or anti-persistent behavior.

**Figure 4:**
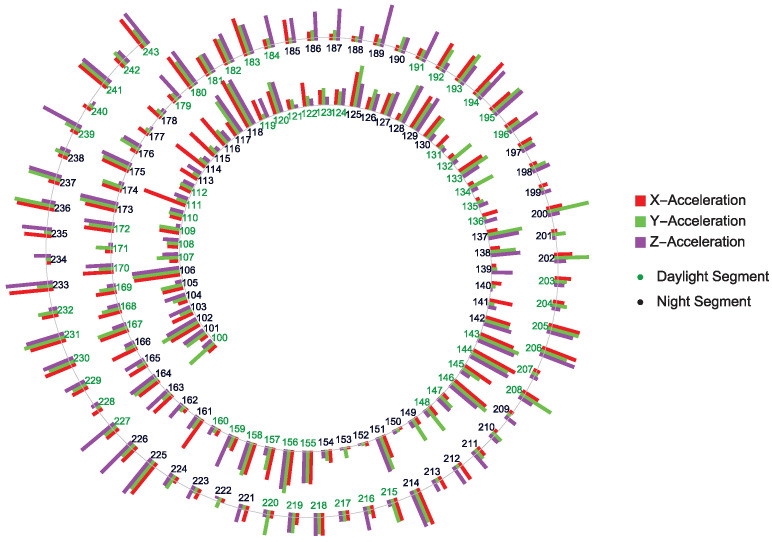
The plot shows the Hurst exponent changes during day and night in 120-min segments of *X*, *Y*, and *Z* complexity projection of the right-hand acceleration during the last approximately 12 days out of 20 days measured. The longer the bars, the closer the patients are to reaching depressive states and to manic phases otherwise. The bars with length approximately in between those extremes, the closer the patient BD episodes are to “normal” Wiener-like time sequences generated by the acceleration of his right hand. The data visualization corresponds to BD patient C207.

A global view of BD evolution spanning about 20 days is shown in Figure 4. The acquired data are segmented into 120 min with a one-minute frequency of data collection. The plot displays quasi-periodic, non-chaotic patterns of, e.g., depression periods represented by the longer bars at, e.g., time segments 225–227, 235–237, etc.

The different complexity distribution of the different direction acceleration points shown in Figure 4 points to possible multifractal BD structure(s) hidden in the actigraphy data.

### 2.3. Predictability

**Figure 5:**
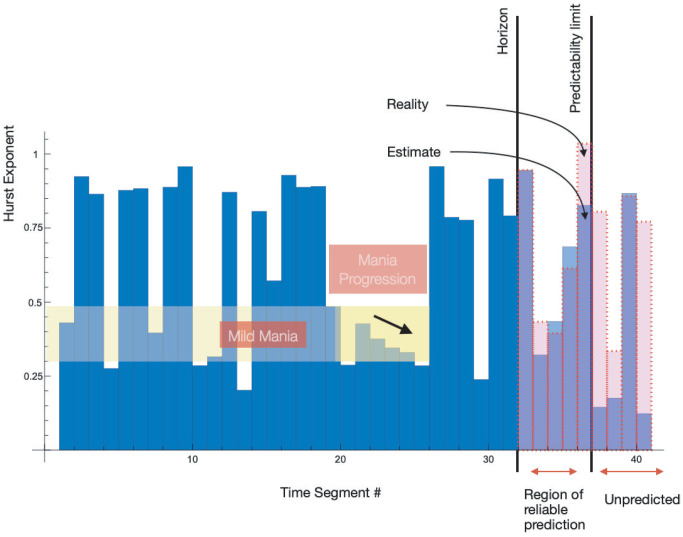
The plot shows application of the presented variational principle to predict the possible evolution of mental deceases and BD in particular. The known data are shortened, and the extrapolation is compared to the real complexity image of patient C207. The Hurst Exponent evolution is shown for the dataset C207 and a predictability evolution of the data. The plot is based on 41 Hurst exponents related to a partition of the original data. Only 31 data points are considered when computing the extrapolation using the approach described above. The predicted and the assumed unknown dynamics of the mesoscale Hurst indices evolution are compared. The red dotted line highlights the assumed unknown Hurst data points, while the light blue rectangles indicate the computed Hurst exponent evolution prediction. The prediction errors are shown in Table 3.

An example of the application of this stochastic optimization is shown in Figure 5 considering a patient’s complexity data. This example can serve as a mechanism for early warning systems such as the prediction of, e.g., manic relapses.

The approach to predictability fundamentally relies on the coarse-graining of microscopic self-similar vital time sequences. This step that is performed by complexity mesoscale projection of microscopic signals is similar to the step from Langevin dynamics to the Fokker–Planck one.

### 2.4. Observable and Meta-Stable Mental Episodes

**Figure 6:**
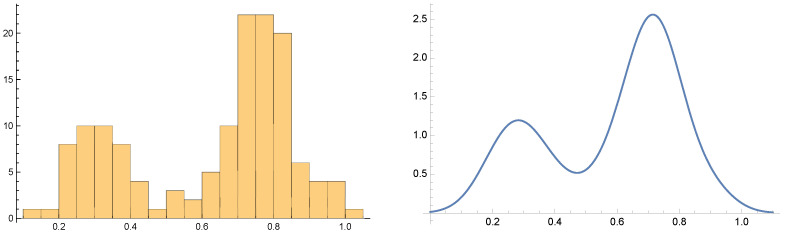
Histogram and the corresponding PDF of mesoscopic (complexity/fractal) projection using the Hurst Exponent of actigraphic data of clinically diagnosed BD patient C207. The PDF exhibits three local extrema. The local minimum corresponds to actigraphy data that can be closely represented by the Wiener unbiased process that we represent as a *stable state*. The local maxima are generated by self-similar timesequences. The lower value, represented by low Hurst exponents, corresponds to more “unpredictable” mental states interpreted as *manic phase*. The other local maximum is, consequently, interpreted as *depression phase* characterized by longer memory data structure repetitions. More details about these data structures are shown at Figure 7.

**Figure 7:**
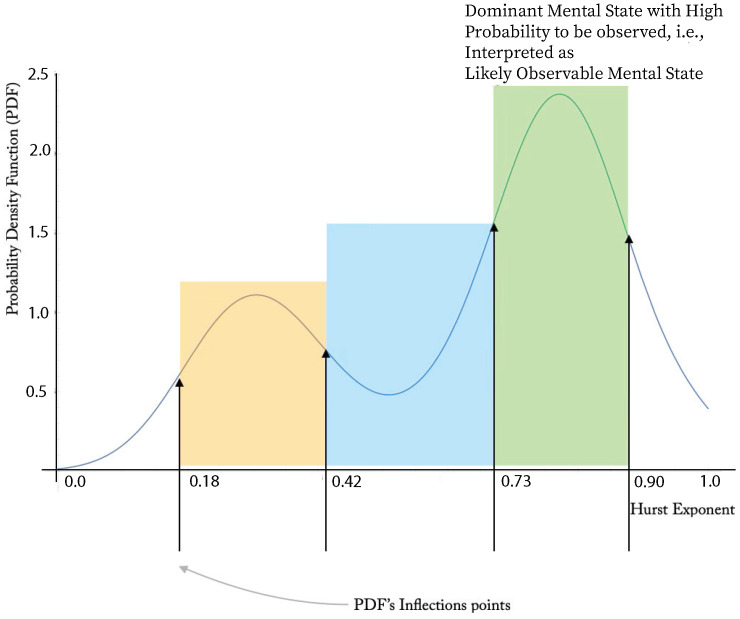
The plot indicates the approximate inflection points, 0.18, 0.42, 0.73, and 0.90 of a smooth PDF corresponding to the (fractal) complexity projection of the actimetry data of patient C207. Particular BD mental states (BD episodes) are determined by the inflection points. The corresponding probability of a particular Mood State(i) is discussed in Section 3.4. The exponent interval (0.73, 0.90) is likely to be the *most* observable state considering dispersive clinical observations that are not based on near-continuous sensor-based objective measurable quantities. The probability values are shown in Table 4. The underlying actimetry-based acceleration data generating the mesoscale PDF appear to be generated by self-similar time sequences. The time-coarsening is based on 120 min data segmentation. The blue region corresponds to an unbiased Wiener process, unlike the other two.

Both the histogram and the PDF shown at Figure 6 resulting from the clinical BD C207 patient actigraphy data exhibit “inverted double-well” structure. We conclude that the BD evolution is a mixture of stable and meta-stable mental states. In general, the propose a stable/meta-stable distinction to be related to the type of local extrema of PDF. Analytically, the state differentiation is computed in the “dual” state of PDF. The approach is demonstrated at Figure 7. The probabilities of the particular states are computed at Table 4.

The probabilities shown at Table 4 point to another important aspect of the presented semicontinuous approach. We indicate at Figure 1 possible overshadowing of BD by an observable depression phase. The actigraphy data corresponding to the C207 patient indicate that the probability of observing the depression episode, 0.34, is higher than the manic and stable episodes. That is likely to mean that a large-in-time dispersion of both subjective and objective evaluations would indicate depression phases, states with a higher observable probability, overlooking the other mental states.

The mathematical aspect of computation of the probabilities shown in Table 4 are discussed in Section 3.4.

The BD episodes probabilities shown in Table 4 correspond to the time distribution of the Hurst exponents displayed in Figure 5. There are about 16 depression episodes in 31 of 120-min segments and 7 manic episodes of the C207 patient. Thus, spared observations will most likely report depression episodes, hiding the manic ones. This conclusion is confirmed by the computed probabilities, c.f., Table 4.

## 3. Mathematical Models

### 3.1. Variational Principle Applicable to Evaluation of the Hurst Exponent

We address fractal dimension estimates of discrete-time sequences using a variational principle that is similar to the approach advocated in [14].

Consider a constitutive law of invariance. Specifically, assume $\mathrm{dist}\{x_{i},\frac{1}{2}\left(x_{i-1}+x_{i+1}\right\}$ to be proportional to a time or spatial resolution via a power law

(1) $$\begin{array}{c} \sum_{i=1}^{n}\mathrm{dist}\{x_{i},\frac{1}{2}\left(x_{i-1}+x_{i+1}\right\}∼\\ a\;\min\{{\left(\mathrm{dist}\left(x_{i},x_{i+1}\right)\;|\;i=1,\ldots ,n\right\}}^{h},\;a\geq 0,\;h\in \left(0,1\right). \end{array}$$

We translate this law as follows. Consider a self-similar time sequence $X\equiv \{x_{i},\;i=1,\ldots$, $2^{m}+1\}$ such that $x_{i}\equiv f\left(t_{i}\right)$ where *f* represents surrogate, possibly sensory, data at times $t_{i}\equiv i\times \frac{T}{2^{m}+1},\;i\in \left(0,2^{m}+1\right)$, $2^{m}+1$ being the total number of complexity, mesoscale, projection of a microscale data time sequence.

Let, on a time interval (0,T),

(2) $${▵}_{m}\overset{\mathrm{def}}{=}\sum_{i=2}^{2^{m}+1}|x_{i}-\frac{1}{2}\left(x_{i-1}+x_{i+1}\right)|.$$

Assume that the equivalent to (1) is

(3) $${▵}_{m}∼a{\left(\frac{T}{2^{m}+1}\right)}^{1-h},\;a\geq 1,\;h\geq 1.$$

Solve the following variational problem to identify the scaling constants $a_{m}$, $h_{m}$ by considering for T>0 given,

(4) $$\begin{array}{cc} \; & \{a_{m},h_{m}\}\equiv \\ \; & \mathrm{ArgMin}\left\{\sqrt{\sum_{k=1}^{m}{\left({▵}_{m}-a{\left(\frac{T}{2^{m}+1}\right)}^{1-h}\right)}^{2}}\;|\;\;a\geq 1,\;h\geq 1\right\}. \end{array}$$

**Figure 8:**
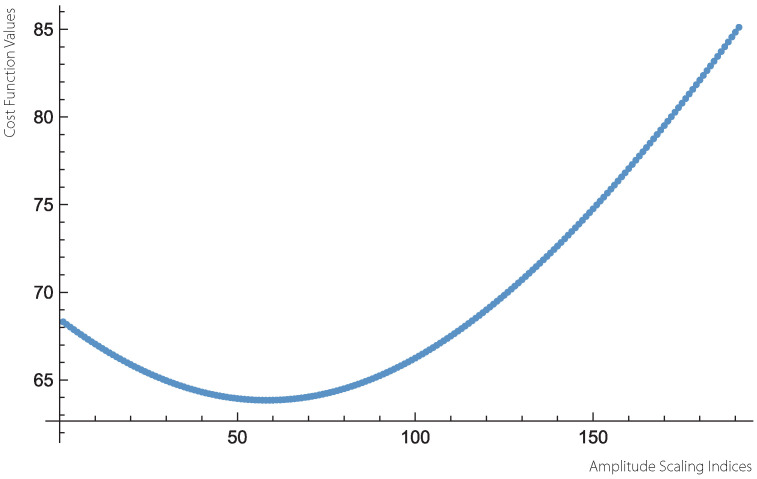
An example of a cross-section plot of the cost function $\sqrt{\sum_{k=1}^{m}{\left({▵}_{m}-a{\left(\frac{T}{2^{m}+1}\right)}^{1-h}\right)}^{2}}$ appearing at (4). The plot represents a fixed value of h=0 and 200 different values of the amplitude scaling parameter *a* in the range (1, 20) with the resolution 0.1. The points appearing in (2) correspond to a fractional white-noise process generating a sequence of 524,288 data points. The cross-section convexity holds for all the values of *a* as well as *h*.

We note the the “cost function” appearing in the variational formulation (4) is convex both in terms of the amplitude scaling parameter *a* as well as the Hurst power parameter *h*, c.f., Figure 8.

#### Examples

We show some approximation capabilities of the method described in Section 3.1 on two examples related to almost nowhere and nowhere differentiable functions that very often model accelerometer-based time sequences. The results summarized by Table 5 and Table 6 represent two of more complex data and their projection on the complexity space in terms of the Hurst exponent.

 **Example 1.**  *Weierstrass Function* (*differentiable on a set of measure zero*)

(5) $$f\left(t,h\right)\equiv \sum_{n=0}^{30}2^{-nh}\cos\left(2^{n}t\right)$$

 **Example 2.**  *Fractional Brownian Motion Process* (*non-differentiable anywhere*)

(6) $$\mathrm{fBm}\left(t,h\right)\equiv \frac{1}{\Gamma \left(h+\frac{1}{2}\right)}\int_{0}^{t}{\left(t-s\right)}^{h-1/2}\;dB\left(s\right),\;\mathrm{where}$$

Γ *is the Gamma function, h is the Hurst index, and B(·) is the Wiener process.*

### 3.2. Stochastic Extrapolation of BD Meso-Scale Data Projection

Consider *n* self-similar stochastic processes X(i) containing *m* complexity mesoscopic projections, H(X(i),m), of its microscopic, possibly sensory, data in domain $\Omega \overset{\mathrm{def}}{=}{\left[0,1\right]}^{n}$, n≥1. The notation H(X(i),m) means that the microscale time sequence is divided into *m* segments, say 129 min, for which the Hurst exponent is computed as described in Section 3.1 and visualized at Figure 3. We construct a Probability Density Function (PDF) for each of the sequences representing complexity representation, H(X(i),m), of the realization of X(i), i.e., we obtain a projection

(7) X(i)→PDF(H(X(i)),m),foreachi≥1.

Consider a process *Y* with complexity projection of its realization ${\left\{y_{i}\right\}}_{i=1}^{k}$, k≪m, and its corresponding PDF(Y,k). Consider the optimization problem

(8) $$i_{0}\equiv \mathrm{Argmin}\left\{{∥\mathrm{PDF}\left(Y,k\right)-\mathrm{PDF}\left(X\left(i\right),m\right)∥}_{L^{p}\left(\Omega \right)},\;i=1,\ldots ,n\right\}.$$

Finally, construct an extended process *Z* by the union of the original realization *Y* and its optimized extension $X(i_{0})$. Specifically,

(9) $$Z\overset{\mathrm{def}}{=}\left\{y_{i=1}^{k}\right\}\cup \left\{x_{i_{0}}{\left(j\right)}_{j>k}^{m}\right\}.$$

A different approach is considered in [15].

#### Identification of Mental Episodes

Assume a diffusional structure of the Hurst exponent evolution. This assumption is important because it is enabled by the presence of mood-persistence models using the Hurst exponent (This approach is similar to the Black–Scholes model used in financial mathematics. The difference is the absence of Dirac-like arbitrary changes in mental disorders. The considered approach resembles more porous media diffusion or apparent diffusion modeling of a cancer treatment-related drug absorption). We propose an extrapolation strategy as follows.

Consider the Fractional Gaussian Noise Process as a choice used to build a library of possible mesoscale scenarios. First, compute the variance of the original Hurst exponents, X, CardX=n, while considering the mesoscale mean to be zero. Second, determine the PDF of the original Hurst Exponent evolution using its mesoscale resolution. Third, build a microscale library of stochastic processes Y,CardY=m,m>n, using FGNP with exponents $h_{i}\in \left(0,\;1\right),\;\mathrm{with}\;i\gg 1$. The number of considered data time-sequence points is an extended number of the original Hurst exponent mesoscale resolution. Fourth, solve

$$\mathrm{Arcmin}\left\{\mathrm{PDF}\left(X\right)-\mathrm{PDF}\left(Y_{i}\right){L^{p}}_{\left(\Omega \right)}\;|\;i=1,\ldots ,m\right\},\;p>1.$$

**Figure 9:**
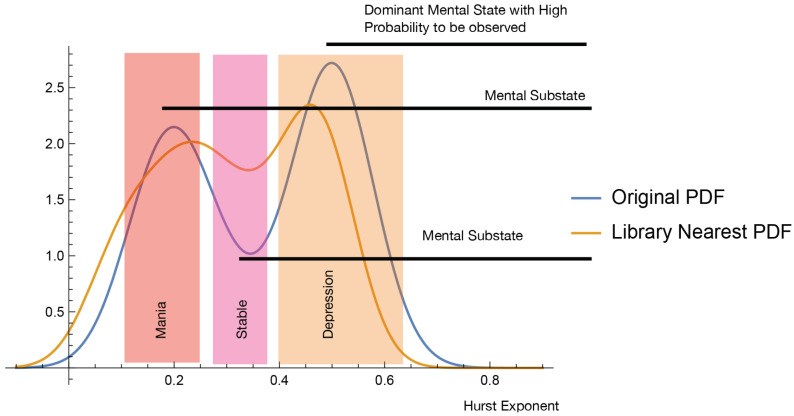
A PDF pronunciation of layered BD structure is detected by the presence of the three local extrema. The orange PDF correspond to a classification model based on 150 library scenarios detecting a specific form of BD. This approach is used to predict evolution as well as recurrence of mental states using objectively measured signs and symptoms. The blue PDF curve corresponds to the data of patient C207.

An example of the application of this procedure is shown in Figure 9 using BD patient activity analytic data. This procedure also carries a promise of predicting mania relapses, which is one of the unsolved problems.

### 3.3. Evolution of the Mood Change

Consider the evolution of the derivative of the interpolated evolution of the Hurst exponent as an indicator of a mood evolution in terms of decay or increase of the Hurst exponent.

Let X be the Hurst exponent sample. Let f[X](·) be Hermite polynomial interpolation of a complexity sample. Consider the following scenarios shown in Table 7 and in Figure 5 that indicate a mood change from a stable to manic-like behavior after the time segment # 20 indicated the arrow pointing to time evolution.

### 3.4. Computation of the Bipolar Disorder States Probability

Consider a change in BD mental phase inflection points of the (differentiable) PDF representing the mesoscale complexity projection of the self-similar actigraphy time sequences. The patient C207 data shown in Figure 6 indicate multiple extrema of such complexity-based representation. We propose to consider the inflection points to be instances of mental phase changes.

Assuming a smooth PDF, the complexity inflection instances, $x_{i}$, are given by

(10) $$Complexity Inflection Points\overset{\mathrm{def}}{=}\left\{x_{i}\;|\;\frac{d^{2}}{dx^{2}}PDF\left(x_{i}\right)=0,\;i=1,\ldots ,n\right\}.$$

Compute

(11) $$Mood State\left(i\right)\overset{\mathrm{def}}{=}\int_{x_{i}}^{x_{i+1}}PDF\left(x\right)\;dx,\;i=1,\ldots ,n-1,$$

define

(12) $$Mood State\left(\mathrm{Likely}\;\mathrm{Observable}\;\mathrm{BD}\;\mathrm{Episod}\right)\overset{\mathrm{def}}{=}\max\left\{Mood State\left(i\right),\;i=1,\ldots ,n-1\right\}.$$

Considering a BD patient with the code name C207, the plots in Figure 5 and Figure 7 and Table 4 indicate that the patient’s depressive states are most observable at around the Hurst exponent equal to 0.9, with the number of states given by n=3. Moreover, Figure 7 allows the identification of the time segments when high-probability states occur.

## Figures and Tables

**Table 1:** Complexity-based indexing of BD classifies the evolution of repetitive patterns of an observed one-dimensional surrogate time series as a measure of partial brain activity.

Hurst Exponent Range	Behavioral Persistency	Interpretation
0–1/3	Low	Mania
1/3–1/2	Medium	Mild Mania
1/2–2/3	Elevated	Stable
2/3–1	High	Depression

**Table 2:** Young Mania Rating Scale (YMRS) versus objective complexity indexing of BD and their comparison at the days 1, 5, and 10 of the clinical treatment of a BD patient C207.

YMRS	Interpretation	H: x-Accel	H: y-Accel	H: z-Accel	Average	Interpretation
29	Moderate Mania	0.2393	0.1927	0.2122	0.21	Higher Agitation
16	Possible Mania	0.1821	0.2711	0.2373	0.23	Agitation
12	Possible Mania	0.3721	0.2873	0.2927	0.32	Moderate Activity
Correlation						57.76

**Table 3:** Summary of the extrapolation shown at Figure 5 in terms of the errors of the Hurst Exponent estimate. The table also indicates both the mathematical and observational interpretations.

Segment #	Hurst Exponent Estimate	Estimate Error (in %)	Autocorrelation	Interpretation
33	0.945	0.395	Low	Depression
34	0.433	1.290	Medium	Initial prodrom
35	0.395	0.508	Short	BD
36	0.613	0.600	Medium	Pre-depression
37	0.835	1.007	Long	Depression Origin

**Table 4:** The probability of BD episodes shown in Figure 7 of a BD patient C207 computed as discussed in Section 3.4. Both the manic and depressive episodes are characterized by the local maxima of the PDF. That would indicate that such episodes might be characterized as ^meta^-*stable states* as opposed to Wiener processes occurring in the blue region shown in Figure 7 surrounding local minimum.

Meta-Stable: Mania	Stable	Likely Observable: Depression
0.22	0.30	0.34

**Table 5:** Approximation of the Hurst exponent in (5) of the Weierstrass function with $2^{22}$ data points.

Fractal Dimension (FD)	Hurst Exponent	FD Approximation	Error
1.1	0.9	1.01	0.09
1.3	0.7	1.22	0.08
1.5	0.5	1.44	0.06
1.7	0.3	1.65	0.05
1.9	0.1	1.87	0.03

**Table 6:** Approximation of the Hurst exponent in (6) of a Fractal Brownian Motion with $2^{22}$ data points with mean 0 and variance 1.

Fractal Dimension (FD)	Hurst Exponent	FD Approximation	Error
1.1	0.9	1.14	0.04
1.3	0.7	1.32	0.02
1.5	0.5	1.44	0.06
1.7	0.3	1.65	0.05
1.9	0.1	1.85	0.05

**Table 7:** Phenomenological assessment of mood evolution.

Time Derivative	Persistency Change	Interpretation
$\frac{d}{dt}f\left[X\right]\left(t\right)<0$	Memory pattern decay	Manic states expected
$\frac{d}{dt}f\left[X\right]\left(t\right)=0$	Stable	No mood change
$\frac{d}{dt}f\left[X\right]\left(t\right)>0$	Memory pattern elongation	Depressive states expected

**Figure A1:**
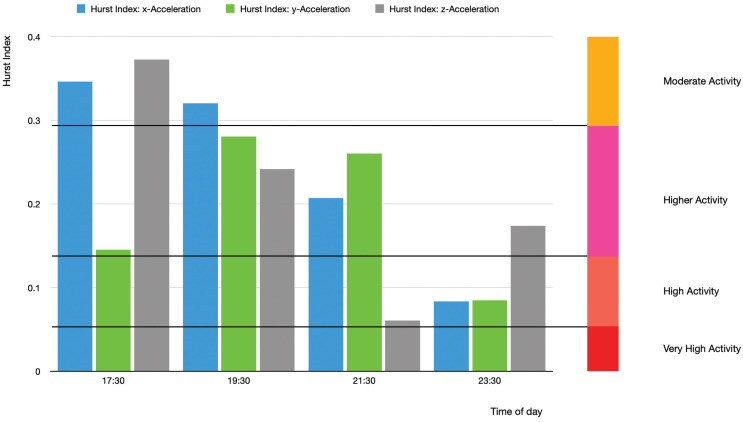
The bar graph corresponds to the results shown at Table A1. The plot seems to confirm the clinical diagnosis of mania.

**Figure A2:**
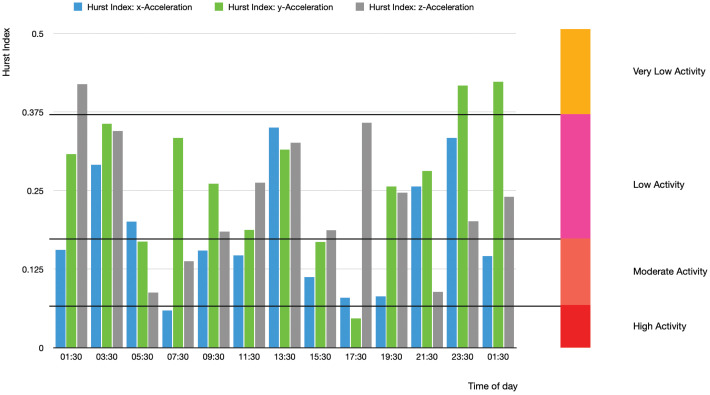
Graphical representation of the data presented by Table A2.

**Figure A3:**
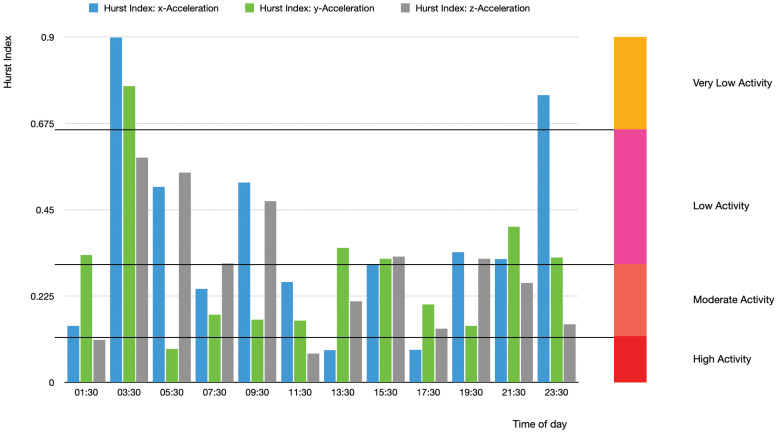
The bar chart corresponds to the data shown at Table A3.

## Data Availability

Data presented can be obtained from the authors upon request.

## Appendix A

### Example of Clinical Application

Subject C207 is clinically in a clear manic state from 17th to 21st of November 2017 and appears to improve from then on, c.f., Table A3. Collecting the complexity indices, confirms the clinical diagnoses in more detail. The considered data do not show any clear periods of depression from a clinical point of view. The data presented in the Table A1, Table A2 and Table A3 were collected in the year 2017.

**Table A1:** The first batch of accelerometer data corresponding to subject C207 represented by their Hurst exponents upon verification of their self-similarity. Complexity based Explanation: Moderate to Higher activity. Evaluation: Negative correlation, i.e., unpredictability. YMRS 29 means Moderate mania.

Day 1	YMRS	Day/Night	Start	End	H(x-Accel)	H(y-Accel)	H(z-Accel)	Mean
11/17	29	Day	15:31	17:30	0.3464	0.1455	0.3726	0.2881
		Day	17:31	19:30	0.3204	0.2807	0.2417	0.2809
		Day	19:31	21:30	0.2070	0.2601	0.0604	0.1789
		Night	21:31	23:30	0.0834	0.0847	0.1738	0.1140
**Mean**					0.2393	0.1927	0.2122	
**Variance**					0.0108	0.0065	0.0128	

**Table A2:** The table indicates five days into the medicated treatment compared to the data of Table A1 and bar chart Figure A1. The Hurst exponent is computed using 120 min segments. Complexity based Explanation: Moderate activity. Evaluation: unpredictability. YMRS 16 means Possible mania.

Day 5	YMRS	Day/Night	Start	End	H(x-Accel)	H(y-Accel)	H(z-Accel)	Mean
11/21	16	Night	23:31	01:30	0.1555	0.3079	0.4195	0.2943
		Night	01:31	03:30	0.2912	0.3561	0.2460	0.3306
		Night	03:31	05:30	0.2006	0.1687	0.0877	0.1523
		Night	05:31	07:30	0.5927	0.3339	0.1375	0.1769
		Day	07:31	09:30	0.1547	0.2614	0.1846	0.2002
		Day	09:31	11:30	0.1469	0.1876	0.2635	0.1990
		Day	11:31	13:30	0.3501	0.3152	0.2630	0.3305
		Day	13:31	15:30	0.1124	0.1684	0.1869	0.1559
		Day	15:31	17:30	0.0794	0.0466	0.3578	0.1613
		Day	17:31	19:30	0.0816	0.2563	0.2460	0.1949
		Day	19:31	21:30	0.2563	0.2815	0.0893	0.2088
		Night	21:31	23:30	0.3337	0.4172	0.2012	0.3117
		Night	23:31	01:30	0.1459	0.4228	0.2403	0.2690
**Mean**					0.1821	0.2711	0.2373	
**Variance**					0.0087	0.0106	0.0099	

**Table A3:** The C207 data confirm an improvement of the patient state upon the applied medication. Complexity based Explanation:: Lower to Moderate Activity. Evaluation: Negative Correlation Unpredictability.

Day 5	YMRS	Day/Night	Start	End	H(x-Accel)	H(y-Accel)	H(z-Accel)	Mean
11/27	12	Night	23:31	01:30	0.1469	0.3331	0.1105	0.1965
		Night	01:31	03:30	0.9899	0.7723	0.3468	0.7523
		Night	03:31	05:30	0.5097	0.0869	0.5468	0.3812
		Night	05:31	07:30	0.2433	0.1762	0.3101	0.2432
		Day	07:31	09:30	0.5207	0.1631	0.4726	0.3855
		Day	09:31	11:30	0.2611	0.1610	0.0751	0.1657
		Day	11:31	13:30	0.0839	0.3511	0.2116	0.2155
		Day	13:31	15:30	0.3057	0.3232	0.3278	0.3186
		Day	15:31	17:30	0.0846	0.2027	0.1391	0.1424
		Day	17:31	19:30	0.3397	0.1475	0.3222	0.2699
		Day	19:31	21:30	0.3213	0.4062	0.2589	0.3288
		Night	21:31	23:30	0.7485	0.3255	0.1511	0.4083
**Mean**					0.3721	0.2873	0.2927	
**Variance**					0.0595	0.0307	0.0264	

## References

[1] Ferrari A.J., Stockings E., Khoo J.P., Erskine H.E., Degenhardt L., Vos T., Whiteford H.A. (2016). The prevalence and burden of bipolar disorder: Findings from the Global Burden of Disease Study. Bipolar Disord..

[2] Fritz K., Russell A.M.T., Allwang C., Kuiper S., Lampe L., Malhi G.S. (2017). Is a delay in the diagnosis 307 of bipolar disorder inevitable?. Bipolar Disord..

[3] Gottschalk A., Bauer M.S., Whybrow P.C. (1995). Evidence of Chaotic Mood Variation in Bipolar Disorder. Arch. Gen. Psychiatry.

[4] Grande I., Berk M., Birmaher B., Vieta E. (2016). Bipolar disorder. Lancet.

[5] Maina G., Adami M., Ascione G., Bondi E., Berardis D.D., Delmonte D., Maffezzoli S., Martinotti G., Nivoli A., Ottavianelli E. (2023). Delphi Panel Collaboration Group; Fagiolini A. Nationwide consensus on the clinical management of treatment-resistant depression in Italy: A Delphi panel. Ann. Gen. Psychiatry.

[6] Teobaldi E., Pessina E., Martini A., Cattaneo C.I., Berardis D.D., Martiadis V., Maina G., Rosso G. (2024). Cariprazine Augmentation in Treatment-Resistant Bipolar Depression: Data from a Retrospective Observational Study. Curr. Neuropharmacol..

[7] Pessina E., Martini A., Raffone F., Martiadis V. (2023). Cariprazine augmentation in patients with treatment resistant unipolar depression who failed to respond to previous atypical antipsychotic add-on. A case-series. Front. Psychiatry.

[8] Krane-Gartiser K., Henriksen T.E.G., Morken G., Vaaler A., Fasmer O.B. (2014). Actigraphic assessment of motor activity in acutely admitted inpatients with bipolar disorder. PLoS ONE.

[9] Heath R.A., Murray G. (2016). Multi-fractal dynamics of activity data in bipolar disorder: Towards automated early warning of manic relapse. Fractal Geom. Nonlinear Anal. Med. Biol..

[10] Mandelbrot B., Van Ness J. (1968). Fractional Brownian Motions, Fractional Noises and Applications. SIAM Rev..

[11] Mörters P., Peres Y. (2010). Brownian Motion.

[12] West B.J. (2010). Fractal physiology and the fractional calculus: A perspective. Front. Physiol..

[13] Kloucek P., Zakharov P., Gunten A. (2016). The Compound Indexing of Human Self-similar Behavioural Patterns. J. Appl. Math..

[14] Liaw S.S., Chiu F.Y. (2009). Fractal dimensions of time sequences. Phys. A Stat. Mech. Its Appl..

[15] Moore P.J., Little M.A., McSharry P.E., Geddes J.R., Goodwin G.M. (2012). Forecasting depression in bipolar disorder. IEEE Trans. Biomed. Eng..

